# *N-*Alkylmorpholines: Potent Dermal and Transdermal Skin Permeation Enhancers

**DOI:** 10.3390/pharmaceutics14010064

**Published:** 2021-12-28

**Authors:** Kristýna Dvořáková, Petr Štěpánek, Jiřina Kroupová, Jarmila Zbytovská

**Affiliations:** 1 Department of Organic Technology, University of Chemistry and Technology Prague, Technická 5, 166 28 Prague, Czech Republic; Kristyna1.Dvorakova@vscht.cz; 2 Department of Chemistry of Natural Compounds, University of Chemistry and Technology Prague, Technická 5, 166 28 Prague, Czech Republic; Petr.Stepanek@vscht.cz; 3 Department of Chemical Engineering, University of Chemistry and Technology Prague, Technická 5, 166 28 Prague, Czech Republic; Jirina.Kroupova@vscht.cz

**Keywords:** dermal and transdermal drug delivery, skin permeation enhancers, skin barrier, morpholine derivatives

## Abstract

Transdermal drug delivery is an attractive non-invasive method offering numerous advantages over the conventional routes of administration. The main obstacle to drug transport is, however, the powerful skin barrier that needs to be modulated, for example, by transdermal permeation enhancers. Unfortunately, there are still only a few enhancers showing optimum properties including low toxicity and reversibility of enhancing effects. For this reason, we investigated a series of new *N*-alkylmorpholines with various side chains as potential enhancers in an in vitro permeation study, using three model permeants (theophylline, indomethacin, diclofenac). Moreover, electrical impedance, transepidermal water loss, cellular toxicity and infrared spectroscopy measurements were applied to assess the effect of enhancers on skin integrity, reversibility, toxicity and enhancers’ mode of action, respectively. Our results showed a bell-shaped relationship between the enhancing activity and the hydrocarbon chain length of the *N*-alkylmorpholines, with the most efficient derivatives having 10–14 carbons for both transdermal and dermal delivery. These structures were even more potent than the unsaturated oleyl derivative. The best results were obtained for indomethacin, where particularly the C10-14 derivatives showed significantly stronger effects than the traditional enhancer Azone. Further experiments revealed reversibility in the enhancing effect, acceptable toxicity and a mode of action based predominantly on interactions with *stratum corneum* lipids.

## 1. Introduction

The transdermal application of drugs is an important administration route, providing many benefits, such as the avoidance of the first-pass metabolism, controlled drug delivery over long time periods, and increased patient compliance. The main aim of the transdermal formulation is an adequate drug permeation rate through the skin to the bloodstream [1,2]. On the contrary, in dermal delivery, the major target is the skin tissue where sufficient drug concentrations are needed. However, for both transdermal and dermal deliveries, the skin protective function hinders the entrance of external substances into the organism and therefore represents a fundamental obstacle. The main skin barrier is located in its uppermost layer, the *stratum corneum* (SC) [3,4]. SC has a special arrangement described as the “brick and mortar model”, where the “bricks” represent corneocytes, the terminally differentiated epidermal cells filled with keratin. The “mortar” is the lipid matrix of characteristic composition (ceramides, cholesterol and free fatty acids of various lengths) [5]. This special arrangement ensures the essential barrier function but also limits the diffusive transport of drugs [6,7,8].

One of the methods of overcoming the skin barrier, to increase the penetration of drugs, is the application of skin permeation enhancers [9]. These substances increase the drug flow through the SC, mainly by one or more of the following mechanisms: (i) interactions with SC lipids, (ii) interactions with SC proteins and (iii) increasing the drug solubility in the vehicle and affecting its partitioning in the SC [10,11]. Besides a high efficacy, permeation enhancers are required to be non-irritating, non-toxic and non-allergenic. They should not possess their own pharmacological activity, their effect must be reproducible and predictable, and the changes they induce in the skin should be reversible [12,13]. From the regulatory point of view, enhancers are defined as pharmaceutical excipients and should fulfil particular qualitative specifications, including the ability to provide their intended functionality and reversibility of their effect or impact on the adhesive properties of transdermal patches [14,15].

Although many different structures interacting with the SC have been evaluated for their ability to enhance drug permeation via the skin, only a few compounds meet all the qualitative demands. Moreover, most of the enhancers are not universal and are effective for only a limited number of drugs. Therefore, the scale of permeation enhancers should be rather broad to ensure an optimum drug/enhancer combination for each particular formulation. Efficient methods of skin permeation enhancement are essential also from the commercial point of view. Currently, to approve a new transdermal product on the market, it must provide substantial clinical benefits like significantly higher drug bioavailability compared to analogous oral formulations [11]. All of these facts inspire scientists to search for new structures showing high enhancing activity and the other required properties.

Many potent permeation enhancers are amphiphilic—they possess a polar and non-polar part in their molecule. Such enhancers favourably solubilise the drug and simultaneously penetrate the SC lipid lamellae. Their polar heads interact with the polar regions by breaking the hydrogen-bonding network and their hydrophobic chains incorporate between the chains of SC lipids, causing a disturbance of the rigid lipid packing, lateral fluidisation and a decrease of the skin barrier resistance. Such amphiphilic structure can be found in Azone-, amino acid- and sugar- derivatives, carbamates, terpene derivatives and others [7,16]. Amphiphilic enhancers often reveal a bell-shaped relationship between the enhancing activity and their hydrocarbon chain length with maxima at 10–12 carbons for saturated and 18 carbons for unsaturated analogues [11].

An amphiphilic character can also be found in morpholine and thiomorpholine derivatives, which were employed several times as skin permeation enhancers [17,18,19,20]. The alkylmorpholine structure can be derived from the established enhancer *N*-lauryl caprolactam (Azone) by substituting the seven-member ring with the hexane one. It was postulated that the dodecyl morpholine derivatives, in particular, can work as highly potent enhancers and that the morpholine structure is worth investigating [21,22].

This study aimed to synthesise new types of morpholine derivatives as potential skin permeation enhancers. Compared to the structures studied earlier, the hydrocarbon chain in our compounds was not linked by an amide bond. Moreover, we extended the polar head by hydroxymethyl substituents, which could increase the capability to form hydrogen bonds. Our synthetic approach was relatively simple including only two steps. The starting materials were glycosides which are readily available and come from renewable sources.

To investigate their enhancing activity in vitro, we prepared a series of various analogues with saturated (C4-C18) and unsaturated (C18) chains and performed a permeation study using three model permeants of different physico-chemical properties: theophylline (TH), indomethacin (IND) and diclofenac (DF). The most potent enhancers were further evaluated for the reversibility of their effect, mode of action and skin toxicity, using transepidermal water loss (TEWL) measurements, Fourier-transform infrared (FT-IR) spectroscopy and a viability assay study on HaCaT cell line, respectively.

## 2. Materials and Methods

### 2.1. Materials for In Vitro Studies

Permeation enhancers (Table 1) were synthesised in the house (see Supplementary Material). Their partition coefficients LogP were calculated by Chemicalize software (ChemAxon, Budapest, Hungary).

Methanol, acetonitrile, theophylline (TH), diclofenac sodium (DF), indomethacin (IND), gentamicin sulphate and phosphate-buffered saline (PBS) tablets were all purchased from Merck life science (Darmstadt, Germany). Azone—*N*-dodecylazacykloheptan-2-on was purchased from USBiological (Swampscott, MA, USA). Propylene glycol (PG) was purchased from Dr Kulich Pharma (Hradec Králové, Czech Republic). Acetic acid was purchased from Penta (Praha, Czech Republic). All solvents used were of HPLC grade. Water was deionised, distilled and filtered by a Millipore Q purification system.

PBS solution was prepared by dissolving 1 PBS tablet in deionised water (200 mL) at 25 °C to yield a 0.01 M phosphate buffer, pH 7.4.

### 2.2. Synthesis of the Morpholine Derivatives

For general synthetic materials, instrumentation, procedure and characterisation of the prepared compounds, see Supplementary Material.

### 2.3. Effect of Enhancers on Drug Solubility

The effect of enhancers on drug solubility in the donor phase (thermodynamic activity) was determined as follows: 400 μL of the prepared donor sample (see Section 2.5.2) were thoroughly mixed and further incubated for 48 h as in the permeation experiment. Afterwards, the samples were centrifuged for 15 min, 2000× *g* at 32 °C (OHaus, Nänikon, Switzerland) and the supernatant was further diluted with the relevant mobile phase for the particular model drug and analysed by HPLC (see Section 2.4).

### 2.4. High-Performance Liquid Chromatography (HPLC) Analysis

Samples were analysed by a Prominence LC-20 reverse-phase high-performance chromatograph (Shimadzu, Kyoto, Japan), equipped with the following: LC-20AD solvent delivery module with DGU-20A degasser; SIL-20AC autosampler; CTO-20AC column oven; SPD-M20A UV/VIS photodiode array detector; CBM-20A communication module. LCsolution software (1.11 SP1) was used to evaluate the data.

#### 2.4.1. Theophylline

TH was analysed on CC 250/4.6 LICHROSPHER 100 RP 18, 5 µm column (Macherey-Nagel, Germany) at 30 °C with methanol: PBS (pH 7.4) in ratio 40: 60 (*v*/*v*) as mobile phase. The flow rate was 1 mL·min^−1^, injection volume 20 μL and detection wavelength 271 nm. The retention time of TH was 4.2 ± 0.1 min. The column was equipped with a security guard (Phenomenex, Torrance, CA, USA) with SecurityGuardTM Cartridges (C18 4 × 3 mm ID, Phenomenex, Torrance, CA, USA). The TH limit of detection (LOD) was 0.0005 mg/mL and the limit of quantification (LOQ) was 0.002 mg/mL.

#### 2.4.2. Indomethacin

IND was analysed on CC 250/4.6 LICHROSPHER 100 RP 18, 5 µm column (Macherey-Nagel, Germany). The mobile phase consisted of acetonitrile: water: acetic acid 90:60: 5 (*v*/*v*/*v*). The flow rate was 1.5 mL·min^−1^, injection volume 40 μL, column temperature 40 °C and detection wavelength 260 nm. The retention time of IND was 5.0 ± 0.1 min. The column was equipped with a security guard (Phenomenex, Torrance, CA, USA) with SecurityGuardTM Cartridges (C18 4 × 3 mm ID, Phenomenex, Torrance, CA, USA). The IND LOD was 0.001 mg/mL and LOQ was 0.003 mg/mL.

#### 2.4.3. Diclofenac

DF was analysed on CC 150/4.6 KINETEX^®^ 100 RP 18, 5 µm (Phenomenex, Torrance, CA, USA). For the mobile phase, we used methanol: 0.1% acetic acid in water (*v*/*v*) in the ratio of 80:20 (*v*/*v*). The flow rate was 1 mL·min^−1^, column temperature 25 °C, injection volume 20 µL and detection wavelength 279 nm. The retention time of DF was 4.0 ± 0.1 min. The column was equipped with a security guard (Phenomenex, Torrance, CA, USA) with SecurityGuardTM Cartridges (C18 4 × 3 mm ID, Phenomenex, Torrance, CA, USA). The DF LOD was 0.0002 mg/mL and LOQ was 0.0005 mg/mL. All the HPLC methods were previously validated [23,24].

### 2.5. In Vitro Permeation Experiments

#### 2.5.1. Skin Preparation

Porcine earlobes were purchased from a local slaughterhouse. In the first step, hairs were removed by a trimmer. After that, the skin was isolated by a dissector. The thickness of the skin was 1.02 ± 0.04 mm. The obtained skin sheets were washed in PBS and stored at −20 °C for a maximum of 3 months.

#### 2.5.2. Donor Samples’ Preparation

Three drugs were used as model permeants, namely TH (Mw = 180.16 g∙mol^−1^, logP = −0.02, pKa = 8.81; Drugbank, ALOGPS; solubility in PBS = 7.65 mg.mL^−1^ [25]), IND (Mw = 357.79 g∙mol^−1^, logP = 4.27, pKa = 4.5; Drugbank, ALOGPS; solubility in PBS = 0.65 mg.mL^−1^ [26]), and DF (Mw = 318.13 g∙mol^−1^, logP = 4.26, pKa = 4.0; Drugbank, ALOGPS; solubility in PBS = 5.15 mg.mL^−1^ [27]). The donor samples were prepared as 5% (*w*/*v*) suspension of TH, DF and 2.5% (*w*/*v*) suspension of IND; all in 60 % PG in distilled water (*v*/*v*). Some donor samples were used as negative controls. To others, 1 % (*w*/*v*) of the particular morpholine derivative was added. In this part of the study, all the synthesised alkylmorpholines (Table 1) were tested. Azone (1% *w*/*v* in 60% aqueous PG) was used as a positive control. All samples were vortexed several times for 10 min and stored in an incubator at 32 °C for 24 h prior to the permeation experiment. Before application to the skin, the samples were re-suspended by vigorous shaking on the vortex.

#### 2.5.3. Permeation Experiment

To study the activity of the permeation enhancers, Franz-type diffusion cells with a diffusion area of 1 cm^2^ were used. The square fragments of thawed skin were mounted on the diffusion cells, dermal side down and donor and acceptor compartments were clamped together. The acceptor compartment was filled with 5–6 mL of PBS (pH = 7.4) containing 50 mg·L^−1^ of gentamicin, to prevent microbial contamination upon sample storage. The PBS was used for its capability to simulate and maintain physiological pH and isotonicity. The precise volume of each cell was always determined for inclusion in later calculations. The acceptor phase was stirred by a magnetic bar and placed in a water bath thermostated at 32 °C throughout the experiment. After a hydration equilibrium period of 2–3 h (PBS in the acceptor compartment, empty donor compartment), the skin integrity was verified by initial TEWL measurements of intact skins, using a Tewameter^®^ TM 300 probe and a Multi Probe Adapter Cutometer^®^ MPA 580 (C+K electronics, Köln, Germany). Thereafter, 500 mL of PBS was added for 15 min to estimate the skin electrical impedance (see Section 2.6 for more detail). In the next step, the PBS was completely removed and 300 μL of the donor suspensions, shaken on a vortex immediately prior to use, were applied to the skin surface. The diffusion cells were covered by parafilm to minimise evaporation. During the permeation experiments running for 48 h, 300 μL of the acceptor phase was withdrawn in defined time intervals and replaced with the same volume of fresh acceptor buffer solution.

#### 2.5.4. Permeation Evaluation

The cumulative amount (*Q*) of the model drugs which permeated across the skin, was corrected for acceptor phase replacement and cell volume and plotted against time. Steady-state flux (*Jss*) values were calculated as the slope of the linear part of the permeation curves. The potencies of the particular enhancers were expressed as the enhancement ratio (*ER*) of the permeant flux with and without the enhancer according to

(1) $$ER=\frac{J_{{ss}_{enhancer}}}{J_{ss_{control\;}\;}}$$

The permeation coefficients (*K_p_*) of the particular permeants in the skin were calculated according to $K_{p}=\frac{J_{SS}}{c_{0}}$, where c_0_ is the concentration of the permeant in the donor compartment.

#### 2.5.5. Entrapment of API in the Skin

At the end of the permeation experiment, the Franz diffusion cells were dismounted and the skin surface was thoroughly washed by distilled water and PBS. The skin area, which was exposed to the donor phase, was cut out, weighed and extracted by 5 mL of the respective mobile phase by shaking for 24 h at 32 °C on a laboratory shaker. The extracts were then filtered through 0.45 µm cellulose filters to eliminate skin residues and analysed by HPLC. Drug concentration in the skin, expressed as mg of model drugs per g of the skin tissue was calculated by dividing permeants’ amounts by the respective skin weights.

### 2.6. Electrical Impedance Measurements

For monitoring the skin integrity and characterisation of the permeation enhancers’ effect, the electrical impedance of each skin was measured, using an LCR 4080 multimeter (Voltcraft, Hirschau, Germany), set in parallel-equivalent mode with an alternating current frequency of 120 Hz. The measurements were carried out according to established protocols [25,26,28,29,30,31]. The measurements were performed in the Franz diffusion cells. After the initial hydration and equilibration, the donor compartments were filled with 500 μL of PBS. One wire of the multimeter was placed in PBS in the acceptor, the other in the donor phase and the electrical impedance was determined. The next measurement was taken after the 48 h permeation experiment. When the donor samples (300 mL) were removed, they were replaced with 500 μL PBS and the electrical impedance was re-measured. The electrical impedance ratio (*EIR*) was calculated for each cell as

(2) $$EIR=\frac{EI_{1}}{EI_{2}}$$

where *EI_1_* [kΩ] is electrical impedance before and *EI_2_* [kΩ] after the 48 h treatment by the donor formulation.

### 2.7. Transepidermal Water Loss (TEWL) Measurements

The reversibility of the enhancers’ effects was studied by evaluation of the difference in the water loss through the intact and enhancer-treated skin. It was measured using a Tewameter^®^ TM 300 probe and a Multi Probe Adapter Cutometer^®^ MPA 580 (C+K electronics, Köln, Germany). The TEWL measurements were carried out according to established protocols [31,32,33,34,35]. The skin samples were mounted into Franz diffusion cells, with the acceptor compartments filled with PBS pH 7.4 and placed in a water bath at 32 °C to hydrate and equilibrate. At first, the base TEWL values of untreated skin samples were measured. Each of the Franz diffusion cells was removed from the water bath, dried completely with compressed air and inserted into a holder. Then the TEWL probe was placed 1.0 cm above the skin surface. The measuring time was on average 80–100 s. The steady-state values were noted at comparable ambient conditions (air temperature 24–26 °C and relative air humidity 27–46%). After this base TEWL value measurement, 150 μL of 1% of the selected enhancer (Mo10, 12, 14 or Azone) in 60% aqueous PG or of the control (60% aqueous PG with no enhancer) was applied into the donor compartment. The cells were then incubated at 32 °C. The donor samples were removed after 2 h or 24 h and the skin surface was washed with 0.5 mL of PBS, gently blotted dry and thoroughly dried with compressed air. After the enhancer was removed, TEWL was measured at predetermined intervals over 42 or 50 h.

### 2.8. Attenuated Total Reflectance (ATR)–FTIR Measurements

ATR-FTIR is a powerful method to investigate the interactions of enhancers with SC lipids and proteins. The skin was mounted to Franz diffusion cells in a water bath at 32 °C and treated with 180 µL of 1% enhancers (Mo10, Mo12, Mo14, Mo18/1) in 60% aqueous PG. Pure distilled water or 60% aqueous PG were applied as controls. After 24 h exposure, the donor phase was carefully removed, the skin was washed by distilled water and the exposed skin was cut out. IR spectra of the skin surface were recorded by an IR spectrometer (Nicolet iZ10, Thermo Scientific, Waltham, MA, USA), equipped with a single reflection MIRacle ZnSe crystal (PIKE Technologies, Madison, WI, USA) at constant clamping pressure. The spectra were generated by the co-addition of 64 scans, collected at a resolution of 2 cm^−1^ and evaluated by OMNIC™ software (Thermo Scientific, Waltham, MA, USA). Collected spectra were normalised, to reduce potential variations in the overall intensity of the spectra. Selected regions (amide I, amide II and CH_2_ stretching vibration bands) were fitted in Origin Pro (OriginLab, Wellesley Hills, MA, USA). The position, height and area of these selected peaks were compared among the particular spectra and the variances between peak characteristics were statistically evaluated by GraphPad Prism^®^ software (GraphPad Software, Inc., La Jolla, CA, USA).

### 2.9. Toxicity Measurements

Cytotoxicity of the three best enhancers (Mo10, Mo12, Mo14) was evaluated on non-malignant spontaneously immortalised human keratinocytes HaCaT (Cell Lines Service, Eppelheim, Germany). The cell line was cultivated in Dulbecco’s Modified Eagle’s Medium (DMEM, Sigma Aldrich, St. Luis, MO, USA), supplemented with 10% Fetal Bovine Serum (Sigma Aldrich, St. Luis, MO, USA) in a Petri dish at 37 °C in a humidified atmosphere of 5% CO_2_. Cells were subcultured two times a week. For cytotoxicity experiments, 96-well plates were seeded with cells (at a density of 10,000 cells per well) in 100 μL of culture medium and incubated at 37 °C in a humidified atmosphere of 5% CO_2_ for 24 h.

The enhancers were dissolved in DMEM, left in an ultrasonic bath for 15 min and then kept at 37 °C overnight. On the next day, 10 μL of the particular enhancer solution was added to 100 μL of cell suspensions, to receive final enhancer concentrations in the range of 0.9–110 μM (concentrations determined according to our preliminary testing). The cells were incubated with tested compounds for 24 h. Then, the cell viability was determined, using the Cell Counting Kit-8 (CCK-8, Sigma Aldrich, St. Luis, MO, USA). The cells were incubated with 10 μL of the CCK-8 for 2 h. Thereafter, absorbance was measured at 450 nm, using the Infinite 200 PRO microplate reader (Tecan Austria GmbH, Austria). The mean optical density (absorbance) was used to calculate the percentage of cell viability as follows:

$$\mathrm{percentage}\;\mathrm{of}\;\mathrm{cell}\;\mathrm{viability}=\frac{A_{treatment}-A_{blank}}{A_{control\;}-A_{blank}}\times 100\;\%,$$

where *A* is the absorbance. The viability assay was expressed as the concentration of enhancers causing 50% cells’ inhibition (IC_50_).

### 2.10. Statistical Analysis

Statistical analysis was performed with the GraphPad Prism statistical program (La Jolla, CA, USA). The outlier values were eliminated using Grubbs’s test. This test is suitable for a large dataset with a normally distributed population. A one-way ANOVA with Dunnetts’ post-test and t-test were used. The differences were considered significant at *p* < 0.05. Data are presented as means ± SEM (standard error of the mean).

## 3. Results and Discussion

### 3.1. The Morpholine Derivatives Show Significant Enhancing Effects

In this work, we studied seven new morpholine derivatives with various side-chain lengths as potential skin permeation enhancers. The saturated chain lengths were of 4–18 carbons. In addition, an unsaturated derivative with an oleyl chain was included. The effect of the enhancers was investigated on porcine skin, using three model drugs. TH was selected as a relatively small molecule with balanced hydrophilic/lipophilic properties and solubility in the donor phase of 20.02 ± 0.62 mg·mL^−1^. The other model permeants, IND and DF, were larger molecules with higher lipophilicity than that of TH, but they differed in their solubility in the donor phase. IND was only slightly soluble (0.95 ± 0.51 mg·mL^−1^) compared to the well soluble DF (57.77 ± 2.55 mg·mL^−1^). Because of the different physico-chemical properties, the selected compounds are proposed to penetrate the SC by different mechanisms. While small hydrophilic molecules are likely to cross the skin barrier by free volume diffusion, the larger lipophilic compounds prefer a lateral diffusion along lipid bilayers [26,36,37]. According to our previous experience, 60% PG in water was used as the vehicle for the donor samples. Cosolvents like PG or ethanol often act synergistically with amphiphilic permeation enhancers [38]. It was shown that aqueous PG changes the conformation of SC proteins, but does not interact with SC lipids, which are mainly the target of amphiphilic enhancers [26,29]. We aimed to utilise this synergy and support the possible effects of our compounds.

**Figure 1:**
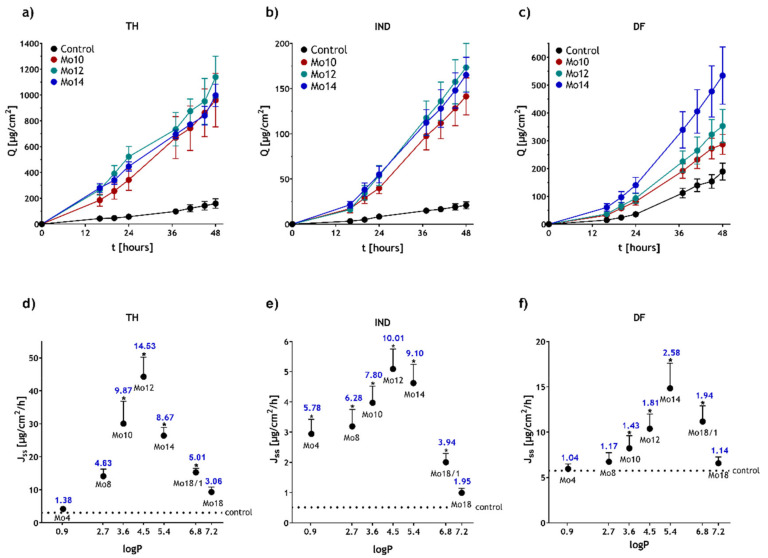
Effects of the studied enhancers (1%) on skin permeability. Permeation profiles of TH (**a**), IND (**b**) and DF (**c**) for the most active enhancers. Flux of TH (**d**), IND (**e**) and DF (**f**) vs. LogP of the studied enhancers. The black dotted line represents the flux of model drugs from a pure vehicle (with no enhancer). The blue numbers represent enhancing ratios. Data are presented as means ± SEM, *n* ≥ 16; * Statistically significant differences compared to the negative control (pure vehicle) at *p* < 0.05.

The results of the permeation study are presented in Table 2. Examples of the permeation profiles of the drugs combined with the most potent compounds are shown in Figure 1a–c. The control TH sample without an enhancer resulted in TH flux of 3.04 ± 0.51 µg·cm^−2^·h^−1^. The IND and DF flux values from the pure vehicle were 0.51 ± 0.09 µg·cm^−2^·h^−1^ and 5.74 ± 1.04 µg·cm^−2^·h^−1^, respectively. Our studies showed that all the morpholine derivatives were efficient as permeation enhancers and able to significantly increase the flux of all three drugs. The most enhancing potency was shown by the morpholine derivatives possessing 10–14 carbons in their side chain. This corresponds to the previously reported data for various structures, stating that chains of middle lengths are the most efficient in enhancement [16,39]. The strongest permeation- enhancing effect for TH and IND was found for Mo12 (ER_Mo12,TH_ = 14.53 and ER_Mo12,IND_ = 10.01). The most potent enhancer for DF was Mo14 (ER_Mo14,DF_ = 2.58). Compared to the positive control Azone (ER 15.34, 1.95 and 4.23, for TH, IND and DF, respectively), the morpholine derivatives show favourable results. Concerning IND, Mo12 has even significantly higher effects than Azone.

The introduction of a double bond into the side chain in Mo18/1 increased its enhancing potency, compared to the saturated derivative Mo18, for all model permeants. This effect was rather expected and was also reported in the literature [13]. The cis-double bond creates a kink in the carbonyl chain that is, therefore, able to interact more efficiently with skin lipids. It is assumed that the oleyl group supports a phase separation and disruption of the otherwise ordered array of saturated straight bands of the lipids [40,41,42]. Interestingly, the unsaturated derivative was overall not the most potent one. Especially in the case of TH and IND, the shorter chain length saturated derivatives Mo10–14 showed a significantly stronger enhancing effect.

In general, our results showed a bell-shaped dependency between the side-chain length in the morpholine structure and the enhancing activity for all the permeants. Similar effects were described previously for Azone derivatives, amino acids, lactate, ceramides derivatives and others [43,44]. It was suggested that shorter chain analogues would provide a more permeable shortcut for penetrating compounds, probably due to the formation of free space within the SC lipid lamellae, which would then be filled by neighbouring chains, causing lateral fluidisation of the tightly packed lamellar arrangement [45]. Naturally, the length of the side chain influences the overall lipophilicity of the molecule. An interesting relationship is therefore shown in Figure 1d–f, where the calculated logP values of the enhancers are related to their enhancing effects. The values of the unsaturated derivative Mo18/1 also fit well into this correlation. These results hint at the importance of lipophilicity of the permeation enhancers; more specifically, at their partition abilities between a hydrophilic and lipophilic phase and, by extension, between a vehicle and the SC lipid matrix as suggested earlier [45].

Considering the solubility of the permeants in the donor phase (c_0_ in Table 2), the TH and DF values were hardly affected by the presence of any morpholine derivative. However, the addition of enhancers increased the solubilities of otherwise sparingly soluble IND, showing a bell-like dependency, approximately correlating with the flux results. This indicates a solubilisation ability of our compounds in particular for less soluble lipophilic drugs. This could contribute to the final enhancing effect and explain why the IND permeation is more enhanced (ER_Mo12, IND_ = 10.01) than that of DF (ER_Mo14, DF_ = 2.58). In any case, this increase in solubility has probably only a minor effect on the overall enhancing activity. The permeation coefficients (K_p_), describing the ability of the permeants to diffuse inside the membrane, were significantly influenced by the enhancers for all the model drugs, corresponding to the bell-like relationships of their flux values. This indicates that the enhancers directly interacted with the skin barrier.

### 3.2. The Morpholine Derivatives Increase the Amount of Drugs Accumulated in the Skin

After the permeation experiment, the amount of the model permeants in the skin tissue was determined in relation to the used permeation enhancer (c_skin_ in Table 2). For all the permeants, the studied enhancers significantly increased the accumulation in the skin compared to the negative control. The highest amount of entrapped drugs were found for enhancement by the middle-chained morpholines (Mo10–Mo14) and also for that containing the oleyl moiety (Mo18/1). The absolutely best dermal targeting effect was reached by Mo12 (for TH 1277.71 ± 133.41 compared to control 214.7 ± 32.20 μg per g of the skin (μg·g_skin_^−1^); for IND 563.20 ± 102.73 compared to control 32.43 ± 6.98 μg·g_skin_^−1^ and for DF 777.36 ± 106.27 compared to 380.10 ± 50.52 μg·g_skin_^−1^). Especially in the case of TH and IND, Mo12 showed comparable or even significantly stronger effects than the positive control Azone. Even though the targeting of DF was the most intensive with the use of Mo12, Mo14 was also able to ensure a high amount of the drug in the skin layers (717.53 ± 153.49 μg·g_skin_^−1^ compared to 380.10 ± 50.52 μg·g_skin_^−1^ of the control). These results indicate that the studied enhancers may show their potential not only in transdermal but also in dermal drug delivery. This is an important finding because the formation of a dermal depot is one way to achieve a constant and intensive influx of a drug into the bloodstream [46].

### 3.3. The Morpholine Derivatives Reduce Skin Barrier Function

**Figure 2:**
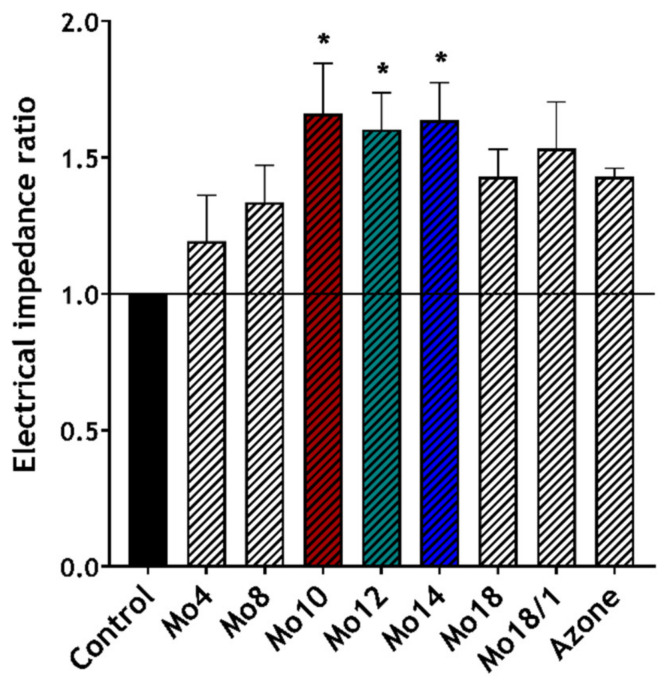
Effects of the studied enhancers (1%) on the skin electrical impedance ratio (EIR). Mean ± SEM, *n* ≥ 12; * Statistically significant difference compared to the negative control.

Electrical impedance (EI) provides important information about the skin barrier integrity and permeability. Therefore, it can be used to characterise direct interactions between enhancers and the skin barrier. In general, higher impedance values mean that the skin barrier is more resistant to the permeation of small charged molecules (e.g., ions) [28,47]. It was shown that EI decreased after the application of permeation enhancers or water [26,34,47]. Figure 2 shows the ratios of the skin electrical impedance (EIR), calculated from the EI measured before and after the enhancers’ application. The use of this ratio provides a clearer means of expressing the enhancers’ influence, without it being hindered by the diversity of the particular skin samples and long hydration times [26,48]. The ratio values for the intact skin control (IND, DF, TH in 60% PG) were set to 1, to emphasise the individual effect of the enhancers according to previous studies [48].

The main increase in the impedance ratio was achieved by the Mo10, 12 and 14 derivatives. Their EIR values of 1.66 ± 0.19; 1.60 ± 0.13; 1.64 ± 0.14, respectively, were significantly higher than that of the untreated controls (impedance ratio value set to 1) and also higher than Azone. These results confirmed that our compounds can interact directly with the skin barrier and reduce its function. Moreover, we also found a slight bell-shaped trend concerning the side chain length, which correlates with our other findings about the enhancers’ effects.

### 3.4. The Morpholine Derivatives Interact with Skin Lipids

IR spectroscopy was applied to study the interactions of the most efficient derivatives (Mo10, Mo12, Mo14) or Azone with the skin barrier, at the molecular level. Moreover, Mo18/1 was also characterised because, contrary to the other derivatives, it possessed the unsaturated side chain which could lead to different interactions. We focused primarily on the region of CH_2_ symmetric (ν_s_CH_2_) and asymmetric (ν_as_CH_2_) stretching vibrations at around 2850 and 2918 cm^−1^, respectively. These bands are related to the arrangement of the lipids in the SC. Low positions of these bands (up to 2850 cm^−1^ for ν_s_CH_2_) indicate higher proportions of trans-conformations and rigidity of the hydrocarbon chains in a crystalline lipid phase. Higher positions of these modes are related to loosening of the rigid arrangement and increased membrane fluidity [49,50]. Table 3 presents the positions of all the studied modes. The skin treated with the pure vehicle (aqueous PG without enhancers) showed ν_s_CH_2_ and ν_as_CH_2_ at 2850.72 ± 0.19 and 2919.36 ± 0.33 cm^−1^, respectively. These positions correlated with previously published results [48,51,52] and indicated relatively well-ordered lipid chains, with predominant trans-conformations in the SC matrix. Shifts of CH_2_-stretching vibrations to higher positions by 1–2 cm^−1^ were observed after the skin exposure to the enhancers. This indicated an increasing proportion of gauche- conformers in the SC lipid chains, which corresponded to a decrease in their order and increasing fluidity. The most significant shifts were observed in skin treated with Mo10 and Mo12. Azone and the unsaturated derivative Mo18/1 also caused an increase in the ν_s_CH_2_ compared to the negative control, though this effect was slighter.

The positions and shapes of the peaks of amide II (1546 cm^−1^) and amide I (1638 cm^−1^) vibrations, which characterise the protein components of the SC, particularly keratin, were also examined. None of the studied enhancers induced significant shifts of the amide modes compared to the control.

Our findings suggest that the main mode of action of the studied enhancers may lay in their interactions with skin lipids. The enhancers increased the fluidity of the SC lipid chains which was documented by the increase in the v_s_CH_2_ and v_as_CH_2_ positions. In particular, the saturated derivatives of decyl- and dodecyl- chains (Mo10 and 12) showed significant changes in the IR-spectra. On the contrary, no changes in the amide region of the SC spectrum indicate that the enhancers do not affect the protein (particularly keratin) conformation in the SC. The observed effects are similar to those described previously for various amphiphilic enhancers [25,34,53].

### 3.5. Effects of the Morpholine Derivatives on the Skin Barrier Are Reversible

**Figure 3:**
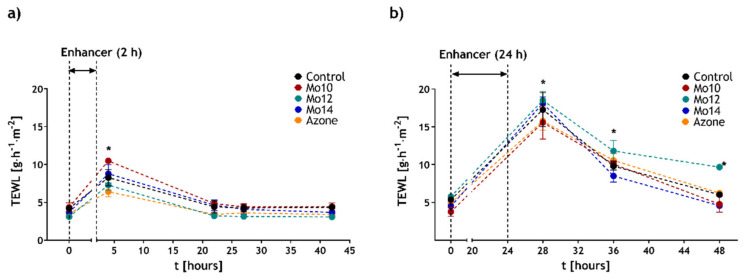
The reversibility of enhancers- effects on skin TEWL when applied for 2 h (**a**) and 48 h (**b**). Mean ± SEM, *n* ≥ 5; * Statistically significant difference compared to the respective control (pure vehicle).

One of the current requirements on permeation enhancers is the reversibility of their effects. This means that the skin barrier can recover its function after the enhancer is removed or has ended its action. To monitor this effect, transepidermal water loss (TEWL) measurements were applied. This method has earlier been confirmed as a suitable tool to detect the reversibility of enhancers’ action in vitro [34,53]. Figure 3 shows the development of TEWL after the enhancers or control acting for 2 or 24 h were removed. The values before the application ranged from 3.1 to 5.8 g·m^−2^·h^−1^. These values were consistent with other studies [54]. After the 2 h–treatment by Mo10, Mo12 and Mo14 the TEWL values increased to 11.7 ± 0.8 g·m^−2^·h^−1^, 8.2 ± 0.3 g·m^−2^·h^−1^ and 9.0 ± 0.4 g·m^−2^·h^−1^, respectively. This increase was significant compared to the respective baseline values. For comparison, the control sample (60 % aqueous PG) at the same time point also showed an increase in TEWL but not significantly different. Later, further to the enhancers’ removal, all the TEWL values slowly decreased to close to the original levels. After 20 h, the difference in TEWL between the treated and control samples was not significant anymore. The values of 4.8 ± 0.1 g·m^−2^·h^−1^, 3.2 ± 0.3 g·m^−2^·h^−1^ and 4.6 ± 0.7 g·m^−2^·h^−1^ for Mo10, Mo12 and Mo14, respectively, were in the same range as 4.4 ± 0.8 g·m^−2^·h^−1^ for the control. Therefore, the effect after a short-term 2 h application of Mo10, 12 and 14 was found to be reversible.

To study the reversibility process after a longer application on the skin barrier, we prolonged the enhancer exposure period to 24 h. After this treatment, the TEWL significantly increased compared to the baseline to 15.6 ± 2.2 g·m^−2^·h^−1^, 18.5 ± 1.2 g·m^−2^·h^−1^ and 18.1 ± 0.8 g·m^−2^·h^−1^ for Mo 10, 12 and 14, respectively. Within the following 24 h after the enhancers’ removal, the TEWL of the skin treated with enhancers dropped to values of 4.3 ± 0.7 g·m^−2^·h^−1^, 9.5 ± 0.1 g·m^−2^·h^−1^ and 4.7 ± 0.1 g·m^−2^·h^−1^, respectively. This did not significantly differ from the control or baseline values before the treatment for Mo10 and Mo14. The TEWL of the skin exposed to Mo12 decreased as well, but more slowly and, even at the end of the experiment, the values were still significantly higher compared to the baseline. The long-term exposure experiment confirmed the reversibility of the enhancers´ effects on the skin barrier, particularly for the Mo10 and Mo14 derivatives. The longer regenerative time for Mo12 can be related to its intensive enhancing function, causing more notable changes in the SC arrangement. Nevertheless, the skin barrier regeneration might be supported by the addition of a renewing agent into the final formulation [48].

To compare our compounds with traditional enhancers, we also treated the skin with Azone according to the same experimental protocol. The development of the TEWL values in time was analogous to the trends of the studied enhancers (Figure 3), confirming the relevance of our derivatives as enhancers with a reversible function.

### 3.6. Cytotoxicity Assay of the Most Efficient Enhancers

**Figure 4:**
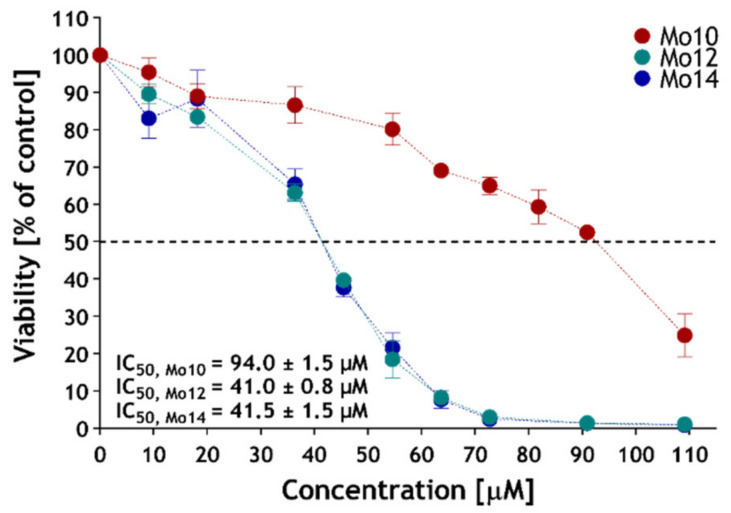
The viability of human keratinocyte cell lines (HaCaT) after 24 h incubation with selected permeation enhancers (Mo10, Mo12, Mo14). Data are presented as means ± SEM, *n* ≥ 4.

The cellular toxicity of the most efficient enhancers (Mo10, Mo12 and Mo14) was studied on the spontaneously immortalised human keratinocytes (HaCaT) cell line. To determine the concentration of the tested enhancers, which cause a 50% decrease in cell viability (IC_50_), CCK-8 was utilised. Figure 4 shows the cell viability in relation to the concentration of the particular enhancers. The IC_50_ of Mo12 and Mo14 was determined at 41.0 ± 0.8 µM and 41.5 ± 1.5 µM, respectively. Interestingly, for Mo10 the value 94.0 ± 1.5 µM was almost double the previous ones. This is consistent with previous studies describing increasing skin toxicity with increasing chain length by various amphiphilic structures [55,56]. The IC_50_ values of our compounds were comparable to or even safer than several enhancers described earlier, e.g., Azone (49 µM [57], 116.9 µM [58]), proline derivative L-Pro2 (68 µM) [29,34], DDAK (76 µM), Transkarbam 12 (21 µM) [29] or dodecyl amino glucoside (24 µM) [34]. This indicates that our compounds show acceptable toxicity and the further potential of application in topical drug delivery.

## 4. Conclusions

In this study, we synthesised novel *N*-alkylmorpholines with various chain lengths and evaluated their activity as skin permeation enhancers. In vitro skin permeation experiments with three different model permeants revealed the high enhancing potency of the prepared compounds. The most efficient morpholine derivatives were those possessing 10–14 chain lengths in both transdermal and dermal delivery. For permeants with higher lipophilicity and low solubility, represented by IND in our study, the *N*-alkylmorpholines showed significantly stronger effects than the established enhancer Azone. The enhancing effect may be supported by the *N*-alkymorpholines´ ability to solubilise IND. Comparable or weaker enhancing effects than those of Azone were achieved for substances with higher solubility (TH, DF) where no solubilisation was observed.

In the subsequent experiments, we focused on the mode of action and safety of the most active enhancers. Using EI and FTIR-spectroscopy, we showed that the main mode of action is probably based on interactions with skin barrier lipids. The fluidity of their hydrocarbon chains increased and the lipids lost their rigid arrangement under the enhancers´ influence. These effects were, however, temporary as confirmed by TEWL. Moreover, the cytotoxicity assay revealed IC_50_ values comparable to other established enhancers. *N*-alkylmorpholines have been shown as promising skin permeation enhancers for local or systemic treatment, meeting the current safety requirements.

Though the potential clinical application of the studied *N*-alkylmorpholines is still disputable, they show benefits that qualify them for further investigation. Their simple synthesis based on available natural sources enables additional functionalisation by other substituents which can be used to further optimise the enhancing effects. In future research, we plan to focus on the structural modifications, optimisation of enhancers formulation, their concentration and, possibly, in vivo studies.

## Figures and Tables

**Table 1:** The structure of the studied morpholine derivatives and their calculated logP values, R = alkyl chain, 18/1 indicates the double bond in position 9.

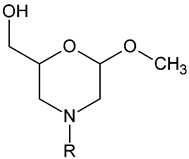	**Enhancer**	**R**	**logP**
	Mo4	C_4_H_9_	0.94
	Mo8	C_8_H_17_	2.72
	Mo10	C_10_H_21_	3.61
	Mo12	C_12_H_25_	4.50
	Mo14	C_14_H_29_	5.39
	Mo18	C_18_H_37_	7.17
	Mo18/1	C_18_H_35_	6.81

**Table 2:** Effects of the studied enhancers (1%) on the permeation of the model drugs applied at 2.5% *w*/*v* (IND) and 5% *w*/*v* (TH, DF) in 60% PG with or without the enhancer. Solubility of model drugs in the donor samples (c_o_), steady-state flux values (J_ss_), enhancement ratio (ER), permeability coefficient (K_p_), the concentration of model drugs in the skin (c_skin_). Data are presented as the means ± SEM, *n* ≥ 16. * Statistically significant difference compared to the negative control (without enhancers) at *p* < 0.05. ^†^ Statistically significant increase compared to a positive control (with Azone) at *p* < 0.05.

TH	c_o_ (mg·mL^−1^)	J_ss_ (µg·cm^−2^·h^−1^)	ER	K_p_ × 10^−4^ (cm·h^−1^)	c_skin_ (µg·mg^−1^)
Control	20.02 ± 0.62	3.04 ± 0.51	-	1.52	214.70 ± 32.20
Mo4	21.36 ± 3.42	4.19 ± 0.56	1.38	1.96	343.40 ± 84.88 *
Mo8	21.19 ± 4.56	14.11 ± 2.15	4.63	6.66	626.30 ± 21.71 *
Mo10	21.19 ± 1.40	30.05 ± 6.78 *	9.87	14.18	814.33 ± 53.07 *
Mo12	20.83 ± 3.30	44.25 ± 5.95 *	14.53	21.24	1277.71 ± 133.41 *
Mo14	20.20 ± 2.73	26.40 ± 2.47 *	8.67	13.07	104.73 ± 88.95 *
Mo18	20.87 ± 2.18	9.32 ± 1.49	3.06	4.46	645.39 ± 44.11 *
Mo18/1	20.67 ± 3.84	15.27 ± 1.22 *	5.01	7.39	685.17 ± 85.83 *
Azone	23.96 ± 2.76	46.71 ± 4.22*	15.34	19.50	1435.43 ± 85.83 *
**IND**	**c_o_ (mg·mL^−1^)**	**J_ss_ (µg·cm^−2^·h^−1^)**	**ER**	**K_p_ × 10^−4^ (cm·h^−1^)**	**c_skin_ (µg·mg^−1^)**
Control	0.95 ± 0.51	0.51 ± 0.09	-	3.75	32.43 ± 6.98
Mo4	4.18 ± 0.41	2.94 ± 0.48 *^†^	5.78	7.02	146.34 ± 34.40 *
Mo8	5.21 ± 0.29 *^†^	3.19 ± 0.56 *^†^	6.28	6.13	222.14 ± 47.68 *
Mo10	5.94 ± 0.42 *^†^	3.97 ± 0.55 *^†^	7.80	6.67	398.07 ± 93.01 *
Mo12	4.90 ± 0.94 *^†^	5.09 ± 0.66 *^†^	10.01	10.39	563.20 ± 102.73 *^†^
Mo14	4.03 ± 1.04	4.62 ± 0.62 *^†^	9.10	11.49	182.36 ± 34.26 *
Mo18	2.86 ± 1.70	0.99 ± 0.15	1.95	3.47	79.23 ± 21.00 *
Mo18/1	2.10 ± 0.85	2.00 ± 0.29	3.94	9.55	158.99 ± 42.45 *
Azone	1.03 ± 0.77	0.99 ± 0.12	1.95	9.60	197.14 ± 45.54 *
**DF**	**c_o_ (mg·mL^−1^)**	**J_ss_ (µg·cm^−2^·h^−1^)**	**ER**	**K_p_ × 10^−4^ (cm·h^−1^)**	**c_skin_ (µg·mg^−1^)**
Control	57.77 ± 2.55	5.74 ± 1.04	-	0.99	380.10 ± 50.52
Mo4	51.13 ± 1.33	5.94 ± 0.55	1.04	1.16	417.01 ± 56.55 *
Mo8	57.88 ± 8.75	6.73 ± 0.99	1.17	1.16	449.36 ± 43.55 *
Mo10	50.76 ± 3.08	8.21 ± 1.39 *	1.43	1.62	651.06 ± 78.35 *
Mo12	57.30 ± 9.62	10.37 ± 1.62 *	1.81	1.81	777.36 ± 106.27 *
Mo14	60.79 ± 6.02	14.82 ± 2.78 *	2.58	2.44	717.53 ± 153.49 *
Mo18	50.12 ± 6.00	6.57 ± 0.68	1.14	1.31	499.29 ± 61.12 *
Mo18/1	54.03 ± 7.78	11.16 ± 1.74 *	1.94	2.06	744.89 ± 138.39 *
Azone	57.00 ± 5.02	24.25 ± 2.47 *	4.23	4.25	1916.98 ± 307.15 *

**Table 3:** Positions of the symmetric and asymmetric CH2- stretching vibrations (ν_s_CH2 and ν_as_CH2, respectively) for the skins treated with selected enhancers measured by ATR-FTIR. Data are presented as means ± SEM, *n* ≥ 9. * Statistically significant difference compared to the respective control (pure vehicle) at *p* < 0.05.

	*v*_s_CH_2_	*v*_as_CH_2_
Control	2850.72 ± 0.19	2919.36 ± 0.33
Mo10	2852.58 ± 0.71 *	2921.91 ± 0.40
Mo12	2851.56 ± 0.23 *	2921.14 ± 0.61
Mo14	2850.74 ± 0.14	2919.48 ± 0.42
Mo 18/1	2850.76 ± 0.15	2920.35 ± 0.36
Azone	2851.16 ± 0.21 *	2921.84 ± 0.34

## Data Availability

Data are contained within the article.

## Supplementary Materials

The following are available online at https://www.mdpi.com/article/10.3390/pharmaceutics14010064/s1; Figure S1: Synthesis of the enhancers; Supporting experimental procedures: Synthesis of the *N*-alkylmorpholines; Characterisation of the prepared compounds.
