# Supplementary material for: N-Alkylmorpholines: Potent Dermal and Transdermal Skin Permeation Enhancers
Source: Pharmaceutics. 2021 Dec 28;14(1):64. doi: 10.3390/pharmaceutics14010064 (PMC8778526; doi:10.3390/pharmaceutics14010064)
Supplement: Supplementary file 1 [file pharmaceutics-14-00064-s001.zip › pharmaceutics-1515413-supplementary.pdf]

pharmaceutics

# Supplementary Materials: N-Alkylmorpholines: Potent Dermal and Transdermal Skin Permeation Enhancers

Kristýna Dvořáková, Petr Štěpánek, Jiřina Kroupová and Jarmila Zbytovská *

## 1. Synthesis of the *N*-alkylmorpholines

The morpholine derivatives included in the study are amphiphilic compounds with a polar head and lipophilic tail. In this study, the polar head is represented by a fragment of the original carbohydrate moiety and the lipophilic moiety by an alkyl chain.

Currently, there are many synthetic procedures by which substances with morpholine ring can be prepared [1]. The chosen approach for the synthesis of the target structures (Figure S1) is based on starting 3-hydroxy-2-(1-methoxy-2-oxoethoxy)propanal, which can be prepared under very mild conditions in multigram scale from *α*-methyl-D-glucopyranoside using the method of Šimák for the respective *β*-anomer [2]. Then, the reductive amination protocol developed by Burland [3] was applied to obtain the morpholine derivatives. This approach is very convenient and versatile for the preparation of libraries of *N*-substituted morpholines.

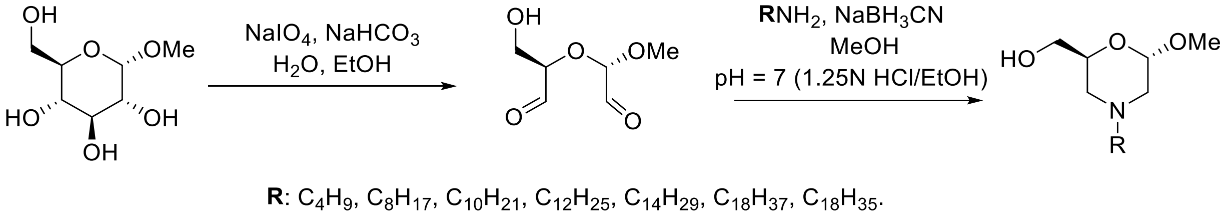


**Figure S1.** Synthesis of the enhancers Mo4 (R –$C_4H_9$, yielded 45%), Mo8 (R –$C_8H_{17}$, yielded 45%), Mo10 (R –$C_{10}H_{21}$, yielded 44%), Mo12 (R –$C_{12}H_{25}$, yielded 31%), Mo14 (R –$C_{14}H_{29}$, yielded 49%), Mo18 (R –$C_{18}H_{37}$, yielded 35%), and Mo18/2 (R –$C_{18}H_{35}$, yielded 29%).

**General Synthetic Methods**. One-dimensional ($^1$H and $^{13}$C) and two-dimensional (COSY, HMQC and HMBC) NMR spectra for final compounds were measured on Bruker AVANCE 500 and 600 instruments ($^1$H at 401 or 600 MHz; $^{13}$C at 125.7 or 150.9 MHz) and Varian Gemini 300 (1H 300 MHz and 13C 75 MHz) as solutions in $CDCl_3$, MeOD 27 °C. Chemical shifts are given in δ (ppm) and coupling constants *J* are given in Hz. HRMS were measured by Micro Q-TOF with ESI ionization. For thin-layer chromatograms were used Stahl plates (10-40 µm, Merck), aluminum TLC sheets-coated silica gel bounded with starch for detection in UV light (Silufol UV 254 nm, Merck) For visualization, vapors of iodine without heating or 50% sulfuric acid in MeOH solution was used and plates were successively heated. For column chromatography, silica gel (32-62 µm, SiliTech, MP Biomedicals and Fluka 60 silica gel was used. All solvents and reagents were obtained from commercial sources and used as obtained unless otherwise noted.

**General procedure of morpholines synthesis.** The starting 3-hydroxy-2-(1-methoxy-2-oxoethoxy)propanal was prepared from methyl-$\alpha$-D-glucopyranoside via methodology of Šimák.[2] Subsequently the obtained dialdehyde was transformed to morpholine following the methodology of Burland.[3] Sodium cyanoborohydride (5 eq) was added to a stirred solution of alkylamine (1 eq), dialdehyde (3 eq), and activated 10 Å molecular sieves in dry methanol. The pH of the reaction mixture was adjusted to 7 with 2 M HCl/$Et_2O$. After stirring for 30 h at room temperature under argon, the reaction mixture was filtered through Célite pad and subsequently concentrated under reduced pressure. The residue was dissolved in ethyl acetate and extracted with brine. The organic layer was dried over $Na_2SO_4$, filtered and concentrated under reduced pressure. The crude residue was purified by column chromatography on silica gel to yield the morpholine derivates.

## 2. Characterization of the prepared compounds

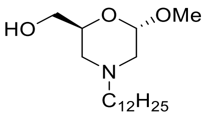


**((2S,6S)-4-Dodecyl-6-methoxymorpholin-2-yl)methanol Mo12.** Column chromatography on silica gel ($CHCl_3$:MeOH 3:0→3:1) yielded 1.8g (31%) of the compound **Mo12** as colorless solid: $^1$H NMR (600 MHz, MeOD) δ 0.90 (3H, t, $J_{12',11'}$ = 7.0 Hz, H12′), 1.23-1.37 (18H, m, H3′-11′), 1.45-1.56 (2H, m, H2′), 1.96 (1H, t, $J_{3a,2}$ = 11.1 Hz, H3a), 2.14 (1H, dd, $J_{gem}$ = 11.7 Hz, $J_{5a,6}$ = 2.8, H5a), 2.24-2.38 (2H, m, H1′), 2.85 (1H, dt, $J_{gem}$ = 11.4 Hz, $J_{3b,2=3b,5b}$ = 2.0, H3b), 2.90 (1H, dt, $J_{gem}$ = 11.7 Hz, $J_{5b,6=5b,3b}$ = 1.5, H5b), 3.38 (3H, s, $OCH_3$), 3.52 (1H, dd, $J_{gem}$ = 11.5 Hz, $J_{CH2,2}$ = 5.0 Hz, $CH_2OH$-a), 3.55 (1H, dd, $J_{gem}$ = 11.5 Hz, $J_{CH2,2}$ = 5.4 Hz, $CH_2OH$-b), 3.96 (1H, bdtd, $J_{2,3a}$ = 10.8 Hz, $J_{CH2,2}$ = 5.2 Hz, H-2), 4.70 (1H, bd, $J_{6,5a}$ = 2.8 Hz, H6); $^{13}$C NMR (150.9 MHz, MeOD) δ 26.84 (C2′), 28.61, 30.47, 30.63, 30.70, 30.71, 30.75, 30.77, (C3′-12′), 33.05 (C1′), 54.92 ($OCH_3$), 55.91 (C3), 56.74 (C5), 60.19 (C1′), 64.25 ($CH_2OH$), 69.90 (C2), 98.06 (C6); HR-MS calcd for $C_{18}H_{38}O_3N$ $[M+H]^+$ 316.28462, found 316.28464. ($CHCl_3$:MeOH 7.5:1) = 0.76.

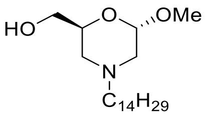


**((2S,6S)-4-Tetradecyl-6-methoxymorpholin-2-yl)methanol Mo14.** Column chromatography on silica gel ($CH_2Cl_2$:MeOH 10:0→10:1) yielded 0.53 g (49 %) of the compound **Mo14** as colorless solid: $^1$H NMR (500 MHz, MeOD) δ 0.89 – 0.95 (3H, t, H14′), 1.31-1.36 (22H, m, H3′-H13′), 1.47 – 1.57 (2H, m, H2′), 1.96 (1H, t, $J_{3a,2}$ = 11.1 Hz, H3a), 2.15 (1H, dd, $J_{gem}$ = 11.7 Hz, $J_{5a,6}$ = 2.8, H5a), 2.32 (2H, m, H1′), 2.86 (1H, dt, $J_{gem}$ = 11.4 Hz, $J_{3b,2=3b,5b}$ = 2.1, H3b), 2.91 (1H, dt, $J_{gem}$ = 11.7 Hz, $J_{5b,6=5b,3b}$ = 1.5, H5b), 3.40 (3H, s, $OCH_3$), 3.48 – 3.62 (2H, m, $CH_2OH$-a,b), 3.98 (1H, bdtd, $J_{2,3a}$ = 10.5 Hz, $J_{CH2,2}$ = 5.2 Hz, H-2), 4.70 – 4.73 (1H, m, H6). $^{13}$C NMR (126 MHz, MeOD) δ 13.43 (C14′), 25.27 (C2′), 29.28, 29.29, 29.30, 29.36, 29.37, 29.41 (C3′-C13′), 31.77 (C1′), 53.54 ($OCH_3$), 54.58 (C3), 55.44 (C5), 58.87 (C1′), 62.78 ($CH_2OH$), 68.62 (C2), 96.81 (C6); HR-MS calcd for $C_{18}H_{38}O_3N$ $[M+H]^+$ 316.28462, found 316.28464. ($CHCl_3$:MeOH 7.5:1) = 0.57.

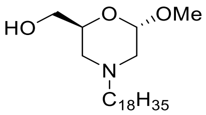


**((2S,6S)-4-*cis*-9′-octadecenyl-6-methoxymorpholin-2-yl)methanol Mo18/1.** Column chromatography on silica gel ($CH_2Cl_2$:MeOH 10:0→10:1) yielded 0.5 g (29 %) of the compound **Mo18/1** as colorless solid: $^1$H NMR (500 MHz, MeOD) δ 0.92 (3H, t, $J_{12',11'}$ = 7.0 Hz, H18′), 1.29 – 1.37 (23H, m, H17′-H11′, H8′-H3′), 1.53 (2H, m, $J$ = 7.7 Hz, H2′), 1.93 – 2.09 (4H, m, H3a, H1′, H8′, H11′), 2.16 (1H, dd, $J_{gem}$ = 11.7 Hz, $J_{5a,6}$ = 2.7 Hz, H5a ), 2.33 (2H, m, H1′), 2.86 (1H, dt, $J_{gem}$ = 11.4 Hz, $J_{3b,2=3b,5b}$ = 2.1 Hz, H3b), 2.92 (1H, dt, $J_{gem}$ = 11.8 Hz, $J_{5b,6=5b,3b}$ = 1.5, H5b), 3.40 (3H, s, $OCH_3$), 3.51 – 3.60 (2H, m, $CH_2OH$-a,b), 3.99

– 4.11 (1H, m, H2), 4.72 (1H, bd, $J_{6,5a}$ = 2.5 Hz, H6), 5.31 – 5.39 (2H, m, H9′, H10′). $^{13}$C NMR (126 MHz, MeOD) δ 13.09 (C18′), 22.38, 26.04, 28.52, 28.64, 28.71, 28.85, 29.02, 29.11, 29.14, 29.17, 29.22, 29.27, 29.29, 29.32, 29.35, 29.37, 29.58, 29.92 (C17′-C11′, C8′-C3′, C2′, C3′, C8′, C11′, C3a), 53.55, 53.93, 54.00, 54.05, 54.97, 55.45, ($OCH_3$, C3, C5, C1′)62.63 ($CH_2OH$), 68.58 (C2), 69.09, 96.77 (C6), 129.44, 129.47, 130.12 (C9′, C10′); HR-MS calcd for $C_{18}H_{38}O_3N$ [M+H]$^+$ 316.28462, found 316.28464. ($CHCl_3$:MeOH 7.5:1) = 0.88.

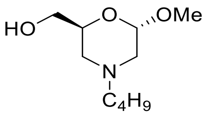


**((2S,6S)-4-Butyl-6-methoxymorpholin-2-yl)methanol Mo4.** Column chromatography on silica gel ($CH_2Cl_2$:MeOH 10:0→10:1) yielded 0.28 g (45 %) of the compound **Mo4** as colorless solid: $^1$H NMR (401 MHz, $CDCl_3$) δ 0.93 (3H, t, $J$ = 7.5 Hz, H-4′), 1.3 (2H, m, H-3′), 1.51 (2H, m, H-2′), 2.08 (1H, t, $J$ = 10.9 Hz, H-3a), 2.20 (1H, m, H-5a), 2.30 – 2.38 (2H, m, H1′), 2.81 (1H, dt, $J$ = 11.3, 2.1 Hz, H-3b), 2.91 (1H, dt, $J$ = 11.6, 1.6 Hz, H-5b), 3.43 (3H, s, $OCH_3$), 3.46 (1H, bs, H-OH), 3.61 (1H, dd, $CH_2OH$-a), 3.70 (1H, dd, $CH_2OH$-b), 4.04 – 4.16 (1H, m, H-2), 4.72 – 4.78 (1H, m, H-6). $^{13}$C NMR (101 MHz, $CDCl_3$) δ 13.91 (C4′), 25.27 (C2′), 28.43 (C3′), 54.58 (C3), 55.05 ($OCH_3$), 55.90 (C5), 58.90 (C1′), 55.44 (C5), 64.06 ($CH_2OH$), 68.37 (C2), 97.21 (C6); HR-MS calcd for $C_{10}H_{21}O_3N$ [M+H]$^+$ 204.15942, found 204.15927. ($CHCl_3$:MeOH 7.5:1) = 0.57.

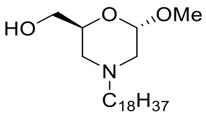


**((2S,6S)-4-Octadecyl-6-methoxymorpholin-2-yl)methanol Mo18.** Column chromatography on silica gel ($CH_2Cl_2$:MeOH 10:0→10:1) yielded 0.43 g (35 %) of the compound **Mo18** as colorless solid: $^1$H NMR (401 MHz, $CDCl_3$) δ 0.83 – 0.94 (3H, t, H18′), 1.27 (30H, m, H3′-H17′), 1.48 – 1.58 (2H, m, H2′), 2.06 (1H, t, $J$ = 10.3, Hz, H3a), 2.19 (1H, dd, H5a), 2.28 – 2.39 (2H, m, H1′), 2.81 (1H, dt, $J$ = 11.1, 2.0 Hz, H3b), 2.91 (dt, $J$ = 11.6, 1.6 Hz, H5b), 3.43 (3H, s, $OCH_3$), 3.62 (1H, dd, $J_{gem}$ = 11.6, $J_{CH2,2}$ = 5.6 Hz, $CH_2OH$-a), 3.71 (1H, dd, $J_{gem}$ = 11.6 Hz, $J_{CH2,2}$ = 3.6 Hz, $CH_2OH$-b), 4.09 (ddt, $J_{2,3a}$ = 11.6 Hz, $J_{2,3b}$ = 3.0 Hz, $J_{CH2,2}$ = 5.9 Hz, H2), 4.75 (1H, m, $J_{6,5a}$ = 1.7 Hz, H6). $^{13}$C NMR (101 MHz, $CDCl_3$) δ 14.23 (C18′), 22.71, 26.17, 27.57, 29.38, 29.43, 29.56, 29.58, 29.62, 29.67, 29.72, 31.94 (C17′-C2′) , 54.18, 55.04, 55.89 (C3, C5, $OCH_3$) 64.22 ($CH_2OH$), 68.76 (C2), 97.19 (C6); HR-MS calcd for $C_{24}H_{49}O_3N$ [M+H]$^+$ 400.37852, found 400.37890. ($CHCl_3$:MeOH 7.5:1) = 0.57.

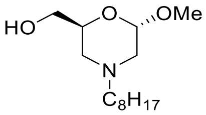


**((2S,6S)-4-Octyl-6-methoxymorpholin-2-yl)methanol Mo8.** Column chromatography on silica gel ($CH_2Cl_2$:MeOH 10:0→10:1) yielded 0.36 g (45 %) of the compound **Mo8** as colorless solid: $^1$H NMR (401 MHz, $CDCl_3$) δ 0.86 – 0.96 (3H, t, $J_{8',7'}$ = 7.1 Hz, H8′), 1.29 (10H, m, H7′-H3′), 1.49-1.58 (2H, m, H2′), 2.11 (1H, m, H3a), 2.17 – 2.28 (1H, dd, $J_{gem}$ = 10.4 Hz, H5a), 2.36 (2H, m, H1′), 2.84 (dt, $J_{gem}$ = 11.3 Hz, H3b), 2.93 (1H, dt, $J_{gem}$ = 11.6 Hz, $J_{5b,6=5b,3b}$ = 1.5 Hz, H5b), 3.44 (3H, s, $OCH_3$), 3.62 (1H, dd, $J_{gem}$ = 11.6 Hz, $J_{CH2,2}$ = 5.6 Hz, $CH_2OH$-a), 3.72 (1H, dd, $J_{gem}$ = 11.6 Hz, $J_{CH2,2}$ = 3.6 Hz, $CH_2OH$-b), 4.11 (1H, m, $J_{2,3a}$ = 10.9 Hz, H-2), 4.76 (1H, bdd, $J_{6,5a}$ = 2.8 Hz, H6). $^{13}$C NMR (101 MHz, $CDCl_3$) δ 14.14 (C8′), 22.68, 26.07, 27.54, 29.22, 29.51, 31.83 (C7′- C2′), 54.18, 55.04, 55.89 (C3, C5, C1′, $OCH_3$) 64.16 ($CH_2OH$), 68.76 (C2), 97.04 (C6); HR-MS calcd for $C_{14}H_{30}O_3N$ [M+H]$^+$ 260.22202, found 260.22208. ($CHCl_3$:MeOH 7.5:1) = 0.58.

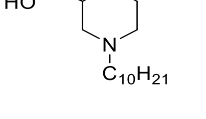


**((2S,6S)-4-Decyl-6-methoxymorpholin-2-yl)methanol Mo10.** Column chromatography on silica gel ($CH_2Cl_2$:MeOH 10:0→10:1) yielded 0.39 g (44 %) of the compound **Mo10** as colorless solid: $^1$H NMR (401 MHz, $CDCl_3$) δ 0.88 (3H, t, $J_{10',9'}$ = 7.4 Hz, H10′), 1.27 (14H, m, H9′-H3′), 1.51 (2H, m, H2′), 2.09 (1H, t, $J_{3a,2}$ = 10.9 Hz, H3a), 2.16 – 2.26 (1H, dd, $J_{gem}$ = 11.7 Hz, $J_{5a,6}$ = 3.1, H5a), 2.28 – 2.39 (2H, m, H1′), 2.83 (1H, dt, $J_{gem}$ = 11.4 Hz, $J_{3b,2=3b,5b}$ = 2.1 Hz, H3b), 2.91 (1H, dt, $J_{gem}$ = 11.6 Hz, $J_{5b,6=}$

$_{5b, 3b}$ = 1.5, H5b), 3.42 (3H, s, $OCH_3$), 3.61 (1H, dd, $J_{gem}$ = 11.6 Hz, $J_{CH2,2}$ = 5.5 Hz, $CH_2OH$-a), 3.70 (1H, dd, $J_{gem}$ = 11.6 Hz, $J_{CH2, 2}$ = 3.7 Hz, $CH_2OH$-b), 4.03 – 4.13 (1H, m, H2), 4.72 – 4.77 (1H, m, H6). $^{13}C$ NMR (101 MHz, $CDCl_3$) δ 14.09 (C10´), 22.67, 26.06, 27.52, 29.31, 29.53, 29.55, 31.89 (C9´-C2´), 54.18, 55.01, 55.80, 59.18 (C3, C5, C1´, $OCH_3$), 64.11 ($CH_2OH$), 68.74 (C2), 97.09 (C6); HR-MS calcd for $C_{16}H_{34}O_3N$ $[M+H]^+$ 288.25332, found 288.25341. ($CHCl_3$:MeOH 7.5:1) = 0.61.

## References


1. Tzara A., Xanthopoulos D., Kourounakis A. P. Morpholine As a Scaffold in Medicinal Chemistry: An Update on Synthetic Strategies *ChemMedChem* **2020**; 15(5), 392–403.
2. Šimák O., Staněk J., Moravcová J. A stereocontrolled synthesis of 3-acetamido-1,3,5-trideoxy- and 1,3,5,6-tetradeoxy-1,5-imino-D-glucitol *Carbohydrate Res.* **2009**; 344(8), 966–71.
3. Burland P.A., Osborn H.M., Turkson A. Synthesis and glycosidase inhibitory profiles of functionalised morpholines and oxazepanes *Bioorg. Med. Chem.* **2011**; 19(18), 5679–92.
